# Precision Oncology: Artificial Intelligence and DNA Methylation Analysis of Circulating Cell-Free DNA for Lung Cancer Detection

**DOI:** 10.3389/fonc.2022.790645

**Published:** 2022-05-04

**Authors:** Ray Bahado-Singh, Kyriacos T. Vlachos, Buket Aydas, Juozas Gordevicius, Uppala Radhakrishna, Sangeetha Vishweswaraiah

**Affiliations:** ^1^ Department of Obstetrics and Gynecology, Oakland University William Beaumont School of Medicine, Royal Oak, MI, United States; ^2^ Department of Biomedical Sciences, Wayne State School of Medicine, Basic Medical Sciences, Detroit, MI, United States; ^3^ Department of Healthcare Analytics, Meridian Health Plans, Detroit, MI, United States; ^4^ Vugene, LLC, Grand Rapids, MI, United States; ^5^ Department of Obstetrics and Gynecology, Beaumont Research Institute, Royal Oak, MI, United States

**Keywords:** DNA methylation, lung cancer, artificial intelligence, machine learning, miRNAs

## Abstract

**Background:**

Lung cancer (LC) is a leading cause of cancer-deaths globally. Its lethality is due in large part to the paucity of accurate screening markers. Precision Medicine includes the use of omics technology and novel analytic approaches for biomarker development. We combined Artificial Intelligence (AI) and DNA methylation analysis of circulating cell-free tumor DNA (ctDNA), to identify putative biomarkers for and to elucidate the pathogenesis of LC.

**Methods:**

Illumina Infinium MethylationEPIC BeadChip array analysis was used to measure cytosine (CpG) methylation changes across the genome in LC. Six different AI platforms including support vector machine (SVM) and Deep Learning (DL) were used to identify CpG biomarkers and for LC detection. Training set and validation sets were generated, and 10-fold cross validation performed. Gene enrichment analysis using g:profiler and GREAT enrichment was used to elucidate the LC pathogenesis.

**Results:**

Using a stringent GWAS significance threshold, p-value <5x10^-8^, we identified 4389 CpGs (cytosine methylation loci) in coding genes and 1812 CpGs in non-protein coding DNA regions that were differentially methylated in LC. SVM and three other AI platforms achieved an AUC=1.00; 95% CI (0.90-1.00) for LC detection. DL achieved an AUC=1.00; 95% CI (0.95-1.00) and 100% sensitivity and specificity. High diagnostic accuracies were achieved with only intragenic or only intergenic CpG loci. Gene enrichment analysis found dysregulation of molecular pathways involved in the development of small cell and non-small cell LC.

**Conclusion:**

Using AI and DNA methylation analysis of ctDNA, high LC detection rates were achieved. Further, many of the genes that were epigenetically altered are known to be involved in the biology of neoplasms in general and lung cancer in particular.

## Introduction

Lung cancer (LC) is the leading cause of cancer deaths in the US and worldwide  (1). There has been a dramatic rise in the incidence of this disorder over earlier decades largely due to smoking and more recently to environmental pollution among non-smokers. The 5-year survival rate is dismal at 4-17% (2) making LC the deadliest cancer in the USA. As per the International Agency for Research on Cancer (IARC) GLOBOCAN cancer statistics, 2.21 million cases of lung cancer cases were diagnosed in the year 2020 and 1.79 million deaths were registered worldwide (3). This high mortality is due principally to the late stage at which most cases are diagnosed highlighting the urgent need for the development of accurate biomarkers.

The US Preventative Services Task Force (USPTF) has recommended routine low-dose computed tomography (LDTC) LC screening of a defined population of high risk individuals (4). The USPTF however found that LDTC screening was associated with harms which included high false positive rates resulting in unnecessary tests and invasive procedures, incidental non-cancerous findings, overdiagnosis and radiation exposure. They therefore called for more research to develop biomarkers to improve the detection rate and lower the false positive rate of LDTC screening (4).

Significant focus has historically been placed on the role of gene mutations in the development of cancer. The extreme variability in the types of gene mutations in cancer however, has made it difficult to develop high sensitivity biomarkers for cancer diagnosis (5) using this approach. The stability and widespread nature of epigenomic changes in cancer has fueled its increasing study for understanding both the pathogenesis of cancer and for novel biomarker development. The best understood and most extensively studied epigenetic change is DNA methylation (6) which can alter gene expression.

### Epigenetics and Cancer

Epigenetics is believed to play a key role in the neoplastic transformation of stem cells to form microscopic benign tumors (7), with extensive increase or decrease of methylation throughout the genome in most and possibly all tumors (8). Many studies have shown that tobacco smoke and other environmental exposures are important in LC pathogenesis, and induce significant epigenetic changes (9–12). Given the extensive degree of methylation changes throughout the genome and the likely role in neoplastic transformation, DNA methylation has great promise as an accurate and early potential biomarker for the detection of cancers.

### Circulating Tumor DNA and LC

‘Liquid biopsy’ involves the harnessing of circulating tumor nucleic acids, such as tumor DNA (ctDNA), micro-RNA, exosomes, and tumor-educated platelets for LC (13) for cancer and other investigations. CtDNA describes cellular DNA released into the bloodstream and is present in higher amounts in cancer compared to normal cases. Several mechanisms such as necrosis and apoptosis induce this DNA release. Furthermore, it is known that newly synthesized DNA is periodically released even from viable intact cells. As a consequence, circulating tumor DNA (ctDNA) has gained increasing attention as a possible source of LC biomarkers (14) both for disease detection and real-time minimally invasive monitoring.

At its core, Precision Medicine deploys a combination of powerful biological approaches (e.g. genomics) and computational and bioinformatic tools for the detection and investigation of complex disorders. Precision Oncology is an established NIH priority (15). We have previously focused on the use of Machine Learning based Artificial Intelligence (AI) and ‘omics’ including epigenomics, metabolomics and proteomics for interrogation of disease mechanisms and the accurate detection of complex disorders (16, 17). Clinically validated DNA methylation markers currently do not exist for LC. In this study we used DNA methylation analysis of ctDNA to interrogate the molecular mechanisms of LC. Further, using multiple AI platforms combined with epigenomic markers, we accurately and minimally-invasively detected LC.

## Materials and Methods

### Study Subjects and Sample Collection

**Figure 1:**
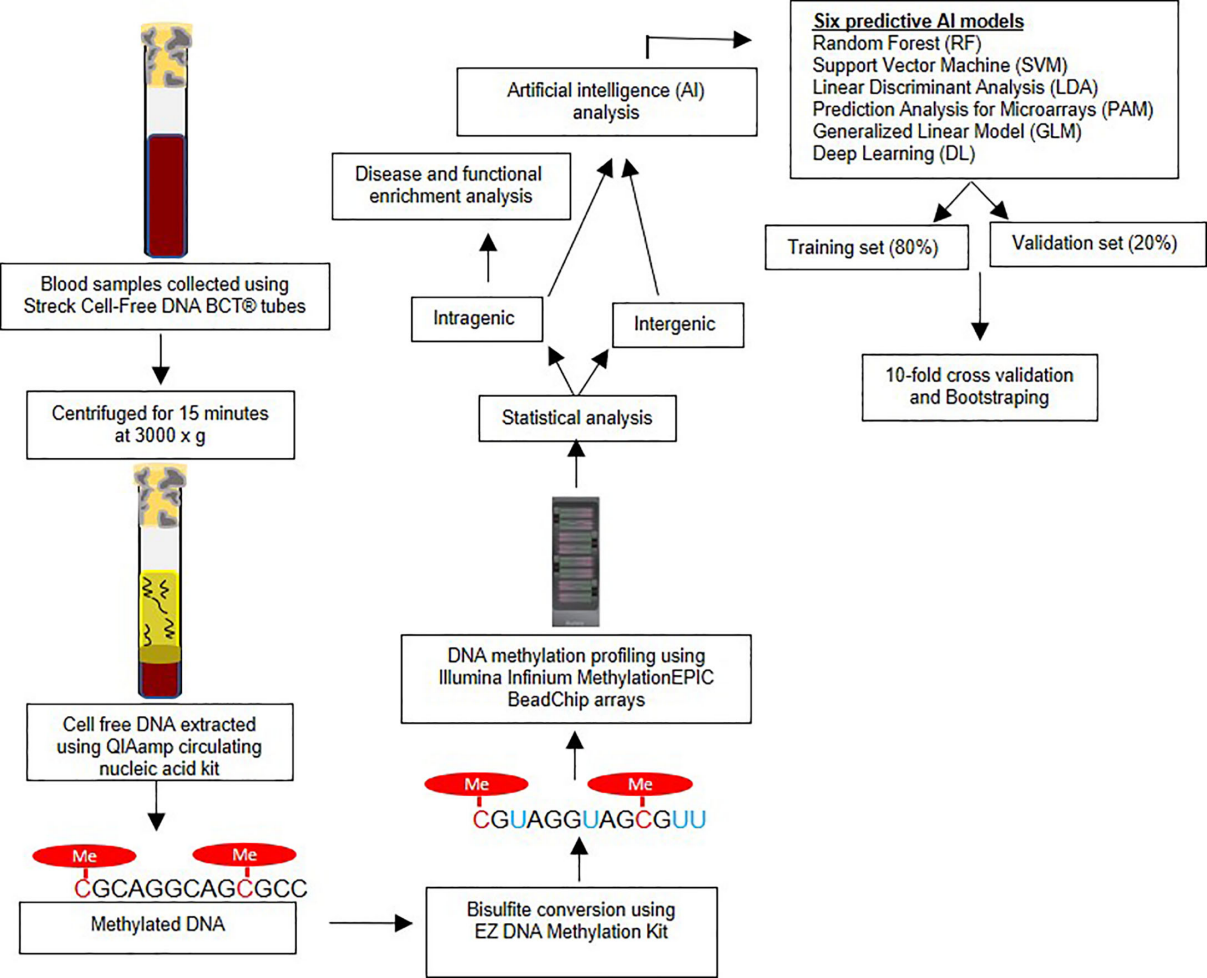
Overview of research methodology – The figure outlines the sample collection, bisulfite conversion, methylation profiling followed by statistical and artificial intelligence analysis.

This study was approved by Beaumont Institutional Review board (IRB#2018-306). Written patient consent was obtained. Blood samples were prospectively obtained from 10 LC cases and 20 controls in the present study. Only cases without any prior treatment for prior or current treatment for lung or other cancers were included in the study. Streck Cell-Free DNA BCT^®^ tubes were used for collecting the blood samples from each study subjects. These tubes are designed to avoid the leukocyte genomic DNA contamination and thus minimizing the dilution and contamination of the cell-free (cf) DNA (18). Medical record numbers were removed, and unique study IDs were allocated to each sample for the purpose of de-identification of samples for laboratory analysis. All samples were processed within 24 hours of sample collection by centrifuging for 15 minutes at 3000 x g and aliquoting plasma into cryogenic vials. Samples were then stored at −80°C until further laboratory analysis (19). The **Figure 1** represents the overview of research methodology including downstream steps considered in the present study.

### Sample Processing and Methylation Profiling

The cf-DNA was extracted using the QIAamp circulating nucleic acid kit (Qiagen Cat # 55114) manual vacuum manifold method. The samples were bisulfite converted using EZ DNA Methylation Kit (Zymo, USA) according to the standardized manufacturer’s protocol. DNA methylation, analysis was performed using the Illumina Infinium MethylationEPIC BeadChip arrays (Illumina, Inc.). The array analyzes approximately 850,000 cytosine (‘CpG’ or ‘cg’) loci covering intragenic and extragenic regions of genome. The assay was performed based on the manufacturer’s protocol, as described in detail previously (20).

### Statistical Analysis

The raw iDat files were analyzed using Illumina GenomeStudio software as described in our earlier studies (20). The β-values (methylation level at each cytosine locus) were measured and compared for statistical differences between the LC and control groups at each cytosine locus using the genome build hg37. To avoid gender bias, the CpG sites on the X and Y chromosomes were not considered in further analyses. Also, CpG loci within 10bp of any Single Nucleotide Polymorphism (SNPs) as observed on Single Nucleotide Polymorphism Database (dbSNP) were excluded as well to avoid genetic (e.g. mutations, single nucleotide polymorphisms) effects on methylation (21). For each CpG marker, the Area Under the Receiver Operating Characteristic (AUC) curve was computed using the R packages dplyr, reshape2 and ROCR. The genome-wide association studies (GWASs) significance p-value threshold < 5x10^-8^ (22) was to designate significant CpG methylation change at each site.

### Artificial Intelligence and Predictive Models for LC Detection

An important aim of our study was to test the performance of AI generated predictive algorithms, consistent with the objectives o Precision Oncology, for the detection of LC. AI ranked the top CpG markers in decreasing order of predictive ability. The top markers were then combined to generate the predictive algorithms for each AI platform. A total of six different AI algorithms were used to as previously reported (17, 23, 24). These platforms were: Random Forest (RF), Support Vector Machine (SVM), Linear Discriminant Analysis (LDA), Prediction Analysis for Microarrays (PAM), Generalized Linear Model (GLM) and Deep Learning (DL). Each has relative strengths and limitations. The data was split into a training set (80% of subjects) and validation set (the remaining 20%) and 10-fold cross validation was performed. The splitting process was repeated ten times and the average area under the receiver operator characteristics curve (AUROC or AUC) and 95% confidence intervals was calculated for LC detection, along with sensitivity and specificity values (25). Bootstrapping using random sampling with replacement was also performed to optimize the accuracy of the estimates. The R package “Caret” was used to optimize predictions for five AI algorithms (RF, SVM, LDA, PAM and GLM) (https://cran.r-project.org/web/packages/caret/caret.pdf), and the package h2o was used to tune the parameters of DL algorithm (https://cran.r-project.org/web/packages/h2o/h2o.pdf) (26–28). The variable importance functions *varimp* in h2o and *varImp* in caret R packages were utilized to rank the models features in each of the predictive algorithms. We used pROC R package to compute the AUC, specificity and sensitivity values of the models (29). The detailed descriptions of AI algorithms, cross validation, bootstrapping, and feature ranking are provided in a **Supplementary Methods Section**.

### Disease and Functional Enrichment Analysis

All analyses were performed using R programming language (v. 4.1.0). The EPIC array CpG loci were annotated using IlluminaHumanMethylationEPICanno.ilm10b4.hg19 Bioconductor package. For each CpG locus we determined the associated gene if any using the UCSC reference gene names (UCSC_RefGene_Name). When multiple genes were associated with a single CpG locus, the most frequently associated gene with that locus was used. Genomic Regions Enrichment of Annotations Tool (GREAT) was used to determine the number of CpGs associated with each gene and the distance of CpGs from the transcription start site (30). CpG methylation changes in transcription start site are more likely to be associated with altered gene expression and therefore to have an identifiable biological effect. g:profiler enrichment was performed using genes associated with statistically significant CpG loci as foreground and all annotated genes as background. R package gprofiler2 (v. 0.2.0) was used to make the enrichment API call with default parameters (31). miRNA enrichment analysis was performed by subjecting significant miRNAs to “miRNA Enrichment Analysis and Annotation Tool” (miEAA) v2.0 (32). We also searched for long non-coding RNA (lncRNA) using “LncExpDB” (33).

### Principal Component Analysis

Given the large number of potential CpG epigenetic predictors generated, dimensionality reduction was performed using Principal Component Analysis (PCA). This approach reduces the number of predictors (dimensionality reduction) and thus simplifies and enhances the interpretability of the data. A visual display is generated showing whether with a limited number of CpG predictors the two groups (LC and controls) can be discriminated. We performed principal component analysis (PCA) MetaboAnalyst (v4.0) (34).

## Results

The demographic details of the study subjects are provided in **Table 1**. All study participants were of Caucasian race. The mean age between two groups was different (Mean age of cases is 64 years and controls were of 75 years, p-value < 0.01), BMI was also lower in LC cases. We therefore performed analysis adjusting for these confounders as well as gender. There were no differences between groups in the frequency of a positive family history for cancer. The histologic types and disease staging of the LC is also presented in **Supplementary Table S1**. Principal component analysis (PCA) showed very good visual the separation of LC and control groups (**Supplementary Figure 1**) using methylation markers. Using the GWAS significance threshold of p-value < 5x10^-8^ (22) we found a total of 4389 CpG loci (intragenic region) (3921 genes) that displayed significant methylation change in LC. Of the total of 4389 CpGs, 2906 were hyper-methylated (increased methylation) and 1483 CpGs were hypo methylated (decreased) in LC compared to control group (**Supplementary Table S2**).

**Table 1:** The demographic characteristics of lung cancer cases and controls.

Parameter	Cases	Controls	p-value
Number of patients	10	20	–
Race - Caucasian	10	20	–
Age - Mean (Standard deviation)	63.9 (11.14)	74.85 (7.37)	0.01 (T)
Gender – n (%)			
Females	7 (70)	14 (70)	0.24 (W)
Males	3 (30)	6 (30)	
BMI - Mean (Standard deviation)	28.9 (3.4)	26.75 (5.3)	0.01 (T)
Family history of any cancer type – n (%)			
Yes	6 (60)	0 (0)	0.09 (W)
No	4 (40)	20 (100)	

T, T test; W, Wilcoxon Mann Whitney test.

We identified 1812 significantly differentially methylated CpGs in non-protein coding region of genome (intergenic region). Among them, 1067 CpGs were hyper methylated and 745 were hypo methylated CpGs (**Supplementary Table S3**). We found that 99% of these CpGs on both intra and intergenic CpGs showed methylation difference of greater than 5%. It should be noted that the higher the methylation difference the more likely is the epigenetic change to correlate with altered gene expression.

### Artificial Intelligence and Lung Cancer Detection

A total of 19 individual CpGs among the intragenic CpGs and four among the intergenic CpGs had an excellent individual predictive value for LC detection based on AUC (AUC =1.00). We performed AI analysis using six different algorithms. Each AI platform was used to rank the CpG markers in decreasing order of predictive ability. We developed separate intragenic (within the gene) and intergenic (based on CpG markers) algorithms for LC detection. Using a 10-marker based algorithm, Five of the 6 AI algorithms using intragenic CpG markers achieved an excellent to outstanding diagnostic performance based on AUC (95% CI). These included SVM, GLM, RF and LDA with AUC=1.00 and 95% CI (0.90-1.00). DL had an AUC (95% CI) =1.00; (0.95-1.00) with 100% sensitivity and specificity, **Table 2**. Bootstrapping yielded excellent predictive accuracies, **Table 2A**. Equal or slightly lower detection rates were achieved when only 5 markers were used. For example, for SVM the AUC (95% CI) =1.00; (0.90-1.00) with 90% sensitivity and 100% specificity and for DL AUC (95% CI) =1.00; (0.95-1.00) with 100% sensitivity and 100% specificity. Likewise, when using 20 markers in the algorithm, the predictive accuracy was slightly higher but generally comparable to the 10-marker model. For example, for SVM the AUC (95% CI) =1.00; (0.90-1.00) with 94% sensitivity and 100% specificity and for DL AUC (95% CI) =1.00; (0.95-1.00) with 100% sensitivity and 100% specificity.

**Table 2:** Artificial Intelligence based prediction on methylation of cf-DNA Lung Cancer for the coding region CpGs (top 10 Variables).

	SVM	GLM	PAM	RF	LDA	DL
AUC 95% CI	1.0000 (0.9000-1)	1.0000 (0.9000-1)	0.9800 (0.8900-1)	1.0000 (0.9000-1)	1.0000 (0.9000-1)	1.0000 (0.9500-1)
Sensitivity	0.9400	0.9700	0.9600	0.9800	0.9200	1.0000
Specificity	1.0000	1.0000	1.0000	1.0000	1.0000	1.0000

CpG predictors in decreasing order of contribution:

SVM: cg06829681 (TEAD1), cg24283889 (LOC102723701; ERLIN2), cg19403339 (DNAJC10), cg01430372 (TMEM99; KRT10), cg23280290 (HERPUD2), cg23178322 (FXR2; SHBG), cg15650170 (AGAP3), cg26864130 (MCAM), cg10299917 (LRP5L), cg25552416 (ZFP3).

GLM: cg10181281 (VWC2L), cg00941912 (KIAA1530), cg21722128 (MEIS3), cg15470857 (ZNF510), cg16267059 (MFAP1), cg16026813 (BTRC), cg25167447 (NAV1), cg16971745 (IFIH1), cg07401887 (DUXAP10), cg13390998 (NFKBIL2).

PAM: cg01430372 (TMEM99; KRT10), cg00071702 (CDH4), cg11149658 (MCPH1), cg07660991 (ZNF414), cg10299917 (LRP5L), cg08855953 (PRKACG), cg18227776 (NCOA2), cg14224170 (SAFB2), cg06270462 (EFHD1), cg00019091 (PTPN11).

RF: cg03871275 (DLK2), cg24847481 (SLC35A3), cg17094927 (ATP8B2), cg07199894 (ULK1), cg06831761 (SRPK2), cg18887033 (CMPK2), cg05398019 (COL27A1), cg24696183 (KCNQ1DN), cg06415550 (PTDSS2), cg16971745 (IFIH1).

LDA: cg26372202 (AK7), cg06819704 (CCNJL), cg10299917 (LRP5L), cg02401627 (LEKR1), cg26864130 (MCAM), cg11107657 (ODZ2), cg26024401 (DCDC2), cg11149658 (MCPH1), cg12282830 (AP1B1), cg25552416 (ZFP3).

DL: cg23496516 (USP36), cg07618979 (NFATC2), cg15684274 (NOC2L), cg06829681 (TEAD1), cg13302670 (CAMK2B), cg21466229 (SNTG1), cg23205538 (PARK2), cg14505733 (WNK2), cg25365034 (KLHL29), cg14364474 (GNAL).

**Table 2A:** Bootstrapping based on methylation of cf-DNA Lung Cancer for the coding region CpGs (top 10 Variables).

	SVM	GLM	PAM	RF	LDA	DL
AUC 95% CI	1.0000 (0.9000-1)	1.0000 (0.9000-1)	0.9822 (0.9000-1)	1.0000 (0.9000-1)	1.0000 (0.9000-1)	1.0000 (0.9500-1)
Sensitivity	0.9500	0.9700	0.9600	0.9800	0.9300	1.0000
Specificity	1.0000	1.0000	1.0000	1.0000	1.0000	1.0000

Likewise, using intergenic (non-coding region of the DNA) CpG markers, SVM, GLM, RF and LDA had excellent to outstanding diagnostic performance with AUC (95% CI) =1.00 (0.90-1.00) and DL performed with AUC (95% CI) =1.00 (0.95-1.00) and 100% sensitivity and specificity **Table 3**. Bootstrapping achieved similar detection performances **Table 3A**.

**Table 3:** Artificial Intelligence based prediction on methylation of cf-DNA Lung Cancer for the non-coding region CpGs (top 10 Variables).

	SVM	GLM	PAM	RF	LDA	DL
AUC 95% CI	1.0000 (0.9000-1)	1.0000 (0.9000-1)	0.9900 (0.8900-1)	1.0000 (0.9000-1)	1.0000 (0.9000-1)	1.0000 (0.9500-1)
Sensitivity	0.9300	0.9600	0.9700	0.9800	0.9400	1.0000
Specificity	1.0000	1.0000	1.0000	1.0000	1.0000	1.0000

CpG predictors in order of contribution:

SVM: cg16349277, cg10302285, cg21127580, cg07591229, cg15455979, cg13645106, cg05458412, cg00316520, cg14185604, cg02475408.

GLM: cg08090691, cg19319928, cg17373554, cg02821627, cg07099084, cg14852082, cg20802868, cg09853648, cg07877987, cg03388189.

PAM: cg05062489, cg09295542, cg06105068, cg24524245, cg19216204, cg03388189, cg06723904, cg13645106, cg12629103, cg02984449.

RF: cg07828654, cg02475408, cg14661028, cg03449513, cg22887498, cg10302285, cg26201011, cg08505243, cg20216928, cg04424605.

LDA: cg24196351, cg14071171, cg14559409, cg07892140, cg12629103, cg10430189, cg06723904, cg05909891, cg09295542, cg17001531.

DL: cg05458412, cg07652774, cg26399254, cg15398272, cg15125549, cg14852082, cg12629103, cg01076051, cg10086080, cg08852943.

**Table 3A:** Bootstrapping based on methylation of cf-DNA Lung Cancer for the non-coding region CpGs (top 10 Variables).

	SVM	GLM	PAM	RF	LDA	DL
AUC 95% CI	1.0000 (0.9000-1)	1.0000 (0.9000-1)	0.9910 (0.9000-1)	1.0000 (0.9000-1)	1.0000 (0.9000-1)	1.0000 (0.9500-1)
Sensitivity	0.9400	0.9650	0.9733	0.9800	0.9475	1.0000
Specificity	1.0000	1.0000	1.0000	1.0000	1.0000	1.0000

**Figure 2:**
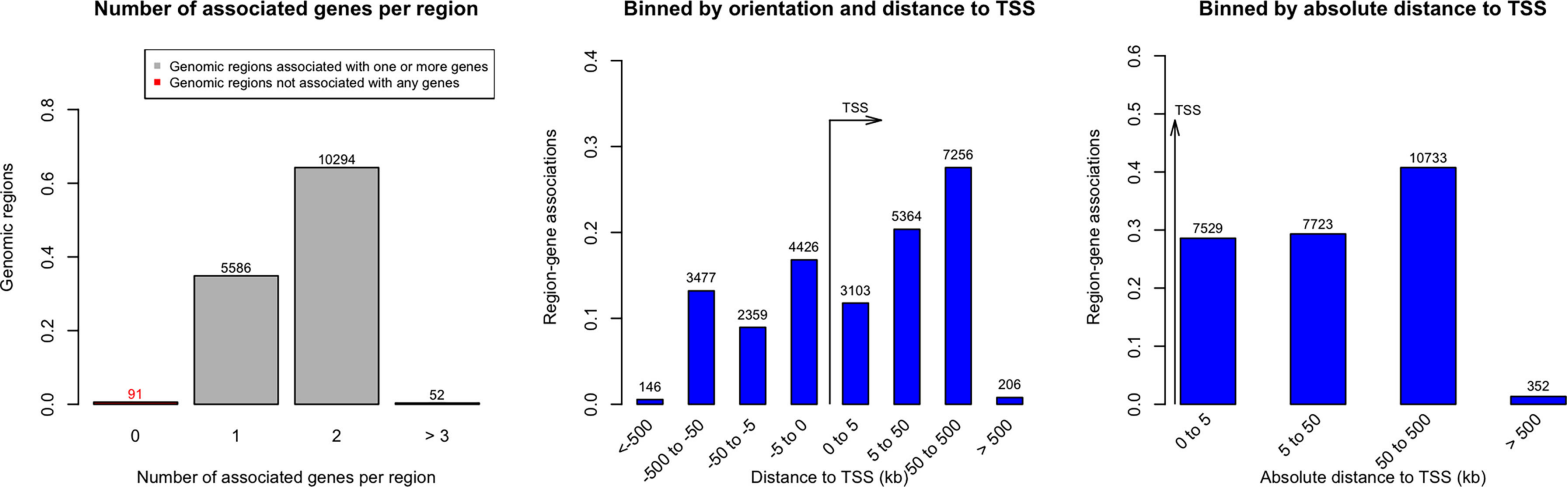
Distance of significantly methylated CpGs’ from Transcription Start Site (TSS) in Lung cancer.

We identified 52 genes with at least 3 of their constituent CpGs significantly differentially methylated, 10294 genes had 2 CpGs and 5586 genes were found to have one CpG that had significant alteration in the methylation level in the ctDNA from LC versus normal group. The orientation of the CpGs from Transcription Start Site (TSS) and absolute distance from TSS are depicted on **Figure 2**. The closer the CpG locus is to the TSS the greater is the likelihood that the methylation change will be biological significant i.e., result in altered gene expression.

Due to the difference in age group of cases and controls, we performed further analysis in which potential confounders such as age and gender were considered with CpG markers. A 50-marker algorithm did not find any of these potential confounders to contribute significantly to LC prediction. All 50 markers for each AI platform were CpG loci for both the intra- and extra-genic analyses (**Supplementary Table S4**).

### Gene Enrichment Analysis

There were 4 significantly enriched terms associated with LC. (1) WikiPathways - Non-small cell lung cancer (NSCLC) (WP : WP4255, p=1.76e-7), (2) KEGG Non-small cell lung cancer (KEGG:05223, p=5.33e-7), (3) WikiPathways - Small cell lung cancer (WP : WP4658, p=0.0020) and (4) KEGG - Small cell lung cancer (KEGG:05222, p=0.0034). The constituent genes in these significantly enriched pathways that were found to be epigenetically altered are listed in **Supplementary Table S5** along with their known or putative roles in LC and in neoplasms in general. Overall, these individual genes based on the quoted references, appear to have a significant role in LC and neoplastic transformation. The epigenetic dysregulation of known LC and cancer molecular pathways lends biological credibility to our findings and supports the argument for a significant role of DNA methylation changes in LC development.

Overall, miRNA genes (epigenetically altered in LC) were found to be enriched and was the top significant term with p-value of <8.24e^-239^ based on g:profiler enrichment analysis. We observed 45 miRNA genes to be significantly differentially methylated in our study. The CpGs (49 CpGs) encompassing regions of these 45 miRNA genes are provided on **Supplementary Table S6** (This is a subset data of **Supplemental Table S2**) and their enrichment status relative to lung cancer is detailed (**Supplementary Table S7**). We also identified 70 CpGs from 66 lncRNA genes that were differentially methylated and associated with LC. The CpGs corresponding to lncRNAs are provided in the **Supplementary Table S8** (This is a subset data of **Supplementary Table S2**). A few of these differentially methylated lncRNAs were previously found to be associated with lung cancer as detailed in the **Supplementary Table S9**.

## Discussion

In 2017 the U.S. Food and Drug administration (FDA) established the Oncology Center of Excellence to promote Precision Medicine in oncology and for the development of new cancer therapies. Its writ included the development of biomarker-based treatments and is grounded in the advances made in our understanding of the genomics of cancer pathogenesis and propagation (35). As noted previously, key to the improvement of LC outcomes will be the development of accurate biomarkers. The potential therapeutic value of liquid biopsies including ctDNA in oncology, have been addressed in other reviews (36). These include cancer screening and diagnosis in asymptomatic populations, identifying individual patients for specific treatments, identifying evidence of residual disease after treatment, predicting the risk of relapse, detection of recurrence, distinguishing true from pseudo progression and reducing prolonged or unnecessary treatments in patients. We combined AI with the DNA methylation analysis of circulating tumor DNA to investigate both the mechanism and for the minimally-invasive detection of LC. We achieved highly accurate detection of LC using six different AI platforms with AUC = 0.90-1.0 and high sensitivity and specificity values. For example, Deep Learning achieved high performance with AUC (95% CI) =1.0, with 100% sensitivity and specificity in this preliminary study. High diagnostic accuracies were similarly achieved with algorithms based on combinations of smaller or larger numbers of individual CpG epigenetic markers. The excellent performance was also achieved when only intragenic or alternatively intergenic CpG loci were considered. In the present study, the classes are moderately imbalanced (i.e., no worse than 10:1). Hence, we did not perform analysis to limit the class imbalance which would otherwise have no huge benefit of considering either weighting or sampling techniques to limit the class imbalance. If there was a class imbalance, we would consider different methods to help improve classification performance. Some of the popular techniques to deal with class imbalances are: (i) Class weights: impose a heavier cost when errors are made in the minority class, (ii) Down-sampling: randomly remove instances in the majority class, (iii) Up-sampling: randomly replicate instances in the minority class and (iv) Synthetic minority sampling technique (SMOTE): down samples the majority class and synthesizes new minority instances by interpolating between existing ones.

We further found evidence of a significant role of epigenetic dysregulation in know molecular pathways involved in LC pathogenesis (discussed in more detail below). Confounders such as age and gender did not appear as independent predictors of cancer beyond the epigenetic markers when we adjusted for these confounders in AI analysis. This is likely due to the fact that these variables have an epigenetic impact which is already subsumed in the DNA data.

Freitas et al. (13) recently reviewed the literature on gene mutation analysis of ctDNA for LC detection. Overall, studies screening for multiple rather than a single cancer gene mutation in ctDNA appeared to have higher diagnostic performance. Gene mutation biomarker studies evaluating a combination from 3 to139 cancer related genes achieved a performance that varied from 33% sensitivity and 100% specificity to a high of 85% sensitivity and 96% specificity.

We focused on DNA methylation given the burgeoning evidence of the centrality of epigenomics in tumorigenesis (37). Other studies have confirmed the feasibility of this approach. Using a combination of methylation markers in 6 cancer genes based on plasma ctDNA analysis, Hsu et al. (38) achieved an 73% sensitivity and 82% specificity for LC detection. Begum et al. (39) performed methylation analysis using serum cell-free DNA. Using a combination of five genes they reported an 75% sensitivity and 73% specificity for LC detection. Zheng et al. (40) achieved a sensitivity of 83.64% and a specificity of 74.0% using a combination methylation profiling of five genes from plasma ctDNA.

Methylation analysis may have a future advantage in facilitating new therapeutic approaches. Targeted alteration of epigenomic changes is emerging as a potentially highly impactful therapeutic approach in cancer. This involves the precise targeting of DNA sequences to reverse or introduce epigenetic marks. The CRISPR/Cas 9 system appears to be the most exciting though not the only such approach (41). The CRISPR/Cas-9 approach has been used for targeted reversal i.e. removal of DNA methylation (demethylation) leading to gene activation in cancer (42).

An important objective of “Precision Oncology” is deploying omics and AI to investigate disease pathogenesis. Recent advances in machine learning (a branch of AI) point to a significant potential for future impact on medical research and practice. It has been noted that AI methods could potentially make significant contributions in the medical field in the following areas: understanding “disease underlying architecture, perform early diagnosis of diseases, and disease progression prediction” (43).

We found alterations in molecular pathways that are involved in non-small cell lung cancer (NSCLC), small cell lung cancer development. Our findings provide further evidence in support of the importance of epigenetic dysregulation in LC. Further, the association with known or suspected LC cancer molecular pathways gives biological plausibility to our findings. The cancer related functions of the genes found to be epigenetically dysregulated in this study is further summarized. In **Supplementary Table S10**, we list the function of genes that were identified to be epigenetically altered and determined by AI to be LC markers, along with their known or suspected roles in LC and neoplastic transformation based on the published literature. Given what is known about their apparent roles of these genes in the neoplastic process, it is therefore not surprising that they emerged as significant markers for LC detection. Examples of epigenetically modified genes that were found in our study and are catalogued in **Supplementary Table S5** include *FHIT, FN1, FOXO3* and *GRB2.* They are thought to regulate epithelial-mesenchymal transition and/or metastasis and associated with LC. Also, *ITGA2, ITGA3* and *ITGA6* are integrin coding genes that participate in cell adhesion, proliferation, and differentiation and are known to have anti-cancer properties in LC. It should be pointed out however that the LC roles of a significant number of genes that were epigenetically altered in our study are currently unknown. Should our findings be subsequently validated, the function of the latter genes in cancer should be investigated. Also, the function of the constituent genes involved in the enrichment pathways reveal an important role in neoplasms in general. Overall, our results were generally enriched with many genes currently known or suspected to be involved in carcinogenesis, giving biological plausibility to our findings.

MicroRNA (miRNA) are small single stranded non-coding RNAs. They play an important role in gene expression through the post- translational regulation of multiple other genes. This is accomplished by binding of miRNA to and degradation of the mRNA of other genes and thus inhibiting their expression. MicroRNA is another well-known epigenetic mechanism. DNA methylation in turn is critical in regulating the expression of miRNA genes (44). miRNA is increasingly being recognized as playing an important role in lung cancer including in tumorigenesis, tumor suppression, with value as biomarkers and potential therapeutic roles among others (45). In the current study, miRNAs overall were found to be significantly enriched, p-value of 8.24e^-249^, in our gene enrichment analysis. We found a total of 45 miRNAs that were significantly differentially methylated and most of them were enriched in various LC phenotypes (**Supplementary Table S6**) signifying the complex regulation of miRNA *via* methylation and regulation of gene expression in LC. Further, we performed a literature review to determine whether our overrepresented miRNAs and their targets were previously identified as having a role in LC pathogenesis. These include miR-96-5p previously identified as an oncogene in lung adenocarcinoma (46), miR-126, miR-212, miR-330, miR-432, miR-563, miR-663a, miR-1238 are considered to be tumor suppressor miRNAs (47–53), miR-136 is significantly upregulated in human NSCLC primary tumors (54). Further, miR-141-3p appears to have prognostic value and is a tumor suppressor involved in the NSCLC progression (55), miR-346 promotes cell growth and metastasis and suppresses apoptosis in non-small cell lung cancer (56), miR-601 is associated with cell apoptosis in lung cancer (57), miR-2861 expression was found to be higher in lung cancer stem cells (58), miR-1307 promotes the proliferation of lung adenocarcinoma (59), the miR-1469 is an apoptosis enhancer that regulates lung cancer apoptosis (60) and miR-200c plays a significant role in suppressing Epithelial-mesenchymal transition in lung cancer (61).

Based on circulating miRNAs studies, the circulating miRNAs, miR-10b and miR141 were found to be elevated in lung cancer cases (62), while circulating miR-487a, miR-30b, miR-601 were found to be associated with NSCLC (63). The serum exosome miR-96 has been identified as a biomarker for lung cancer (64). We also identified lncRNA genes that were differentially methylated in lung cancer and a few of these lncRNAs were already identified in various lung cancer studies (**Supplementary Table S9**).

Although very encouraging, our study is not without limitations. As a proof-of-concept study, the sample sizes were small. it is possible for example that more DNA methylation markers could be detected with the analysis of a larger sample cohort. Despite these limitations, high statistical significance was obtained. Due to the non-suitability of the circulating cell-free DNA for gene expression analyses, we were unable to assess gene expression associated with the methylation changes. We however searched databases based on two studies i.e. 65 (65) and 51 (66) that document gene expression changes in lung cancer tissue. We cross-matched their differentially expressed genes with our differentially methylated genes. We found the following genes to be both differentially expressed in LC tissue and differentially methylated in our study: *DSC3, MUC1, VSNL1, RORC, ACSL5, KRT6B* and *TP63*. Further, many of the CpGs that were epigenetically altered are located close to the gene transcription start site (TSS), which would indicate that methylation changes are likely to impact gene expression. Finally, there were many LC genes with methylation change ≥ 10%. This degree of methylation difference is generally associated with an increased likelihood of gene expression changes (67).

### Conclusion

Using principles espoused in Precision Medicine, we report that a combination of DNA methylation analysis of circulating tumor DNA and AI achieved high LC detection rates based on this minimally invasive approach. High performances were observed with the analysis of either intragenic or intergenic areas of the DNA. In addition, many of the genes that were found to be differentially methylated in LC in our study are known or suspected, based on a search of the existing literature, to be involved in the mechanism of development, suppression, or growth of cancer in general including lung cancer. Larger confirmation studies will need to be performed in the future.

## Data Availability Statement

The original contributions presented in the study are included in the article/**Supplementary Material**. Further inquiries can be directed to the corresponding author.

## Ethics Statement

The studies involving human participants were reviewed and approved by Beaumont Institutional Review Board (IRB#2018-306). The patients/participants provided their written informed consent to participate in this study.

## Author Contributions

RB-S: conceptualization, overview of project, data analysis, and writing manuscript. KV: data analysis and manuscript writing. BA: artificial intelligence methodology. JG: data analysis and enrichment analysis. UR: sample processing, data analysis, and manuscript writing. SV: conceptualization, samples and array processing, data analysis, and writing manuscript. All authors have read and edited the manuscript.

## Conflict of Interest

Author BA was employed by Meridian Health Plans. Author JG was employed by Vugene, LLC.

The remaining authors declare that the research was conducted in the absence of any commercial or financial relationships that could be construed as a potential conflict of interest.

## Publisher’s Note

All claims expressed in this article are solely those of the authors and do not necessarily represent those of their affiliated organizations, or those of the publisher, the editors and the reviewers. Any product that may be evaluated in this article, or claim that may be made by its manufacturer, is not guaranteed or endorsed by the publisher.

## Supplementary Material

Supplementary Figure 1 Principal component analysis (PCA) showing separation of cases and control subjects based on methylation markers in Lung cancer.

Supplementary Table 2 Significantly differentially methylated CpG markers (FDR-p values) for coding regions

Supplementary Table 3 Significantly differentially methylated CpG markers (FDR-p values) for the non-coding DNA regions

Supplementary Table 6 Significantly differentially methylated CpG markers (FDR-p values) for the miRNA coding genes (a subset of **Supplementary Table S2**).

Supplementary Table 7 Over-representation of differentially methylated miRNA in lung cancer phenotypes based on multiple databases.

Supplementary Table 8 Significantly differentially methylated CpG markers (FDR-p values) for the lncRNA coding genes (a subset of **Supplementary Table S2**).
