# Supplementary material for: Precision Oncology: Artificial Intelligence and DNA Methylation Analysis of Circulating Cell-Free DNA for Lung Cancer Detection
Source: Front Oncol. 2022 May 4;12:790645. doi: 10.3389/fonc.2022.790645 (PMC9114890; doi:10.3389/fonc.2022.790645)
Supplement: Supplementary file 1 [file DataSheet_1.docx]

**Supplemental Methods**

**Artificial Intelligence/Deep learning (AI/DL) Analysis Data Preparation**

Missing values were assumed to be the detection limit and substituted with a value that was half of the minimum positive values in the original data. The basis for this assumption is that most missing values are due to low levels of methylation (i.e. below the detection limit). Sample normalization allows general-purpose adjustment for differences between the samples. Data transformation and scaling are two approaches for standardizing and making individual features (predictors) more comparable. The log value of each β-value was centered by its mean (x̅) and auto scaled by its standard deviation (s). Further, quantile normalization method was used to reduce sample-to-sample variation.

**Artificial Intelligence Algorithms**

**Deep Learning (DL):** Deep-learning methods are representation-learning approaches with multiple levels of representation, obtained by composing simple but non-linear modules that each transform the representation at one level (starting with the raw input) into a representation at a higher, slightly more abstract level. With multiple such transformations, very complex functions can be learned. For classification tasks, higher layers of representation precisely targets aspects of the input that are important for group discrimination while suppressing irrelevant variations. This type of hierarchical learning approach is particularly powerful as it allows the program to learn complex representations directly from the raw data (Bengio, 2009). The approach is applicable to multiple disciplines (Goodfellow et al., 2016).

**Random Forest (RF):** This is an increasingly utilized approach. RF generates many classifiers and aggregate their results. Common methods include boosting (Schapire and Yoram, 1998) and bagging (Breiman, 1996) of the classification trees. With boosting, successive trees give extra weight to points incorrectly predicted by earlier predictors. With bagging, successive trees do not depend on earlier trees - each is independently constructed using a bootstrap sample of the data set. RFs add an additional layer of randomness to bagging (Breiman, 2001). In addition to constructing each tree using a different bootstrap sample of the data, RF alters how the classification or regression trees are constructed. In standard trees, each node is split using the best split among all variables. In a random forest, each node is split using the best among a subset of predictors randomly chosen at that node. This approach performs very well compared to many other classifiers and is robust against overfitting (Breiman, 2001). In addition, it has only two parameters (the number of variables in the random subset at each node and the number of trees in the forest) and is generally not very sensitive to their values.

**Support vector machine (SVM):** SVMs (Cristianini and Shawe-Taylor, 2000) algorithms are relatively new. They display significant robustness even in the analysis of limited and noisy data. This has made them a platform of choice for varied applications from text categorization to bioinformatic analysis. SVMs are excellent classifiers, and can separate a given set of binary labeled training data with a hyper-plane that is maximally distant from them (known as ‘the maximal margin hyper-plane’) (Boser et al., 1992). For situations in which linear separation of groups is not possible, SVMs can be combined with the technique of ‘kernels’ that automatically generates a non-linear mapping and separation to a feature space. The hyper-plane found by the SVM in feature space corresponds to a non- linear decision boundary in the input space.

**Linear Discriminant Analysis (LDA):** Principal Component Analysis (PCA) and Linear Discriminant Analysis (LDA) are two commonly used techniques for data classification and dimensionality reduction. Linear Discriminant Analysis easily handles the situations where the within-group frequencies are unequal, and their performances has been examined on randomly generated test data. LDA maximizes the ratio of between-class variance to the within-class variance in a data set thus guaranteeing maximal separation between groups (Balakrishnama and Ganapathiraju, 1998).

**Prediction Analysis for Microarrays (PAM):** is a statistical technique for class prediction using gene expression data using nearest shrunken centroids. The average gene expression level for each gene in each class is determined and divided by the within class Standard Deviation. Thereafter the nearest shrunken centroid classification is calculated. This takes the gene expression profile of a new test group and compares it to each of the class centroids of the previously tested group. The class whose centroid it turns out to be the closest to is predicted to be the class of the new group. The nearest shrunken centroid refers to a further modification by which each of the class centroids is ‘shrunken’ to approach the values of the overall class centroid by a factor that is called the ‘threshold’ value. This is said to improving the accuracy of classification by minimizing the effect of less important contributing genes (Tibshirani et al., 2002). Thus, class prediction is performed on a validation set. This method therefore identifies the subsets of genes that best characterizes and thus discriminates each class.

**Generalized Linear Model (GLM):** The generalized linear models (GLMs) are a broad class of models that include linear regression, ANOVA, Poisson regression, log-linear models etc. But there are some limitations to GLM, such as linear function, which can have only a linear predictor in the systematic component, and responses must be independent.

**Modeling & Evaluation**

Two-step validation was utilized for these analyses. There were two different data sets, first was utilized to build the model and test it, the second one was used to validate the model.

While using the two-step validation method, two different techniques were utilized to find out the best model and calculate the performance metrics: 10-fold Cross validation and Bootstrapping.

1. **10-fold Cross Validation:** The first data set was split into a training to train the model first with a portion of the data and a test group (remaining portion) on which the performance of the developed model is then determined. Here we randomly divided the available set of samples into two parts: a training set and a test or hold-out set. The model was fit on the training set, and the fitted model was used to predict the responses for the observations in the hold-out set. Estimates were used to select best model, and to give an idea of the test error of the final chosen model. Idea was to randomly divide the data into 10 equal-sized parts. We left out part 10, fit the model to the other 9 parts (combined), and then obtained predictions for the left-out 10th part. This was done in turn for each part k = 1, 2...10, and then the results were combined. This process was repeated a total of ten times and the average AUC, sensitivity, specificity and 95% confidence intervals for the test set were calculated. Then, as the validation step, AUC, sensitivity, specificity and 95% confidence intervals for the validation data set were calculated as well (Kim, 2009).
2. **Bootstrapping:** The bootstrap is a flexible and powerful statistical tool that allowed us to use a computer to mimic the process of obtaining new data sets, so that we were able to estimate the variability of our estimate without generating additional samples. Rather than repeatedly obtaining independent data sets from the population, we instead obtained distinct data sets by repeatedly sampling observations from the original data set with replacement. Each of these “bootstrap data sets” was created by sampling with replacement and was the same size as our original dataset. As a result, some observations appeared more than once in each bootstrap data set and some not at all. To estimate prediction error using the bootstrap, we used each bootstrap dataset as our training sample, and the original sample as our test sample. This process was repeated a total of ten times and the average AUC, sensitivity, specificity and 95% confidence intervals for the test set were calculated. Then, as the validation step, AUC, sensitivity, specificity and 95% confidence intervals for the validation data set were calculated (Kim, 2009).

Several parameters were used to tune the models before implementation: Number of trees for RF, classification cost for SVM, threshold amount for shrinking toward the centroid for PAM, and for DL model: a) Epochs (number of passes of the full training set), b) l1 (penalty to converge the weights of the model to 0), c) l2 (penalty to prevent the enlargement of the weights), d) input dropout ratio (ratio of ignored neurons in the input layer during training), e) number of hidden layers. In addition to, l1 and l2 parameters, *input_dropout_ratio* was used as the third parameter to avoid overfitting in DL model which controls the amount of input layer neurons that are randomly dropped (set to zero), controls overfitting with respect to the input data (useful for high-dimensional noisy data). The important step is to randomly drop units (along with their connections) from the neural network during training (Srivastava et al., 2014). This prevents units from co-adapting too much. Using these three parameters, we avoided the biggest complication i.e. overfitting in DL model.

**Ranking Important Features**

The contribution of a feature (predictor) to the model performance was determined using a model-based approach. The importance of each of the features in each of the predictive AI algorithms was ranked by using the variable importance functions *varimp* in h2o and *varImp* in caret R packages.

**References**

Balakrishnama, S., and Ganapathiraju, A. (1998). *Linear Discriminant Analysis—A Brief Tutorial.*

Bengio, Y. (2009). Learning Deep Architectures for AI. *Foundations and Trends® in Machine Learning* 2**,** 1-127.

Boser, B.E., Guyon, I.M., and Vapnik, V.N. (1992). "A training algorithm for optimal margin classifiers", in: *Proceedings of the fifth annual workshop on Computational learning theory.* (Pittsburgh, Pennsylvania, USA: ACM).

Breiman, L. (1996). Bagging predictors. *Machine learning* 24**,** 123-140.

Breiman, L. (2001). Random forests. *Machine learning* 45**,** 5-32.

Cristianini, N., and Shawe-Taylor, J. (2000). *An introduction to support Vector Machines: and other kernel-based learning methods.* Cambridge University Press.

Goodfellow, I., Bengio, Y., and Courville, A. (2016). Deep Learning. *MIT Press*.

Kim, J.-H. (2009). Estimating classification error rate: Repeated cross-validation, repeated hold-out and bootstrap. *Computational Statistics & Data Analysis* 53**,** 3735-3745.

Schapire, R.E., and Yoram, S. (1998). BoosTexter: A System for Multi-Label Text Categorization. *Mars*.

Srivastava, N., Hinton, G., Krizhevsky, A., Sutskever, I., and Salakhutdinov, R. (2014). Dropout: a simple way to prevent neural networks from overfitting. *J. Mach. Learn. Res.* 15**,** 1929-1958.

Tibshirani, R., Hastie, T., Narasimhan, B., and Chu, G. (2002). Diagnosis of multiple cancer types by shrunken centroids of gene expression. *Proc Natl Acad Sci U S A* 99**,** 6567-6572.
