# Supplementary figures and images for: Precision Oncology: Artificial Intelligence and DNA Methylation Analysis of Circulating Cell-Free DNA for Lung Cancer Detection
Source: Front Oncol. 2022 May 4;12:790645. doi: 10.3389/fonc.2022.790645 (PMC9114890; doi:10.3389/fonc.2022.790645)

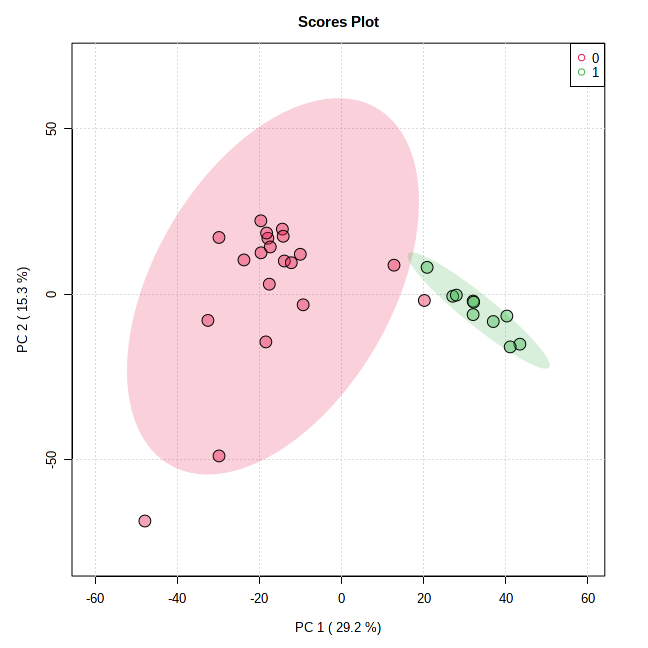

Supplement: Supplementary Figure 1 — Principal component analysis (PCA) showing separation of cases and control subjects based on methylation markers in Lung cancer. [file Image_1.png]
