# Supplementary material for: Precision Oncology: Artificial Intelligence and DNA Methylation Analysis of Circulating Cell-Free DNA for Lung Cancer Detection
Source: Front Oncol. 2022 May 4;12:790645. doi: 10.3389/fonc.2022.790645 (PMC9114890; doi:10.3389/fonc.2022.790645)
Supplement: Supplementary file 3 [file Table_1.docx]

**Supplemental Table S1: Histological characteristics and stag of lung cancer cases**

| Sample | Age at Time of Collection | Gender | Race | BMI | Family Hx CA. | Prior Hx CA. | Histological type CA | Stage CA |
| --- | --- | --- | --- | --- | --- | --- | --- | --- |
| 1 | 47 | Female | Caucasian | 28.17 | No Family hx noted | No pertinent past medical hx. | Poorly differentiated NSCC malignant neoplasm favor adenocarcinoma | pT2a NO |
| 2 | 65 | Female | Caucasian | 30.35 | Father Caner (type not noted) | No pertinent past medical hx. | pulmonary adenocarcinoma well differentiated | pT1a NO M |
| 3 | 72 | Female | Caucasian | 24.6 | Brother Cancer (type not noted), Sister Cancer (type not noted) | No pertinent past medical hx. | Invasive Squamous Cell Carcinoma | pT3 N1a |
| 4 | 55 | Female | Caucasian | 33.63 | Father, Maternal Grandmother, Paternal Grandfather Cancer (type not noted) | No pertinent past medical hx. | Adenocarcinoma of lung primary | pT1bNO |
| 5 | 56 | Male | Caucasian | 29.59 | Father Leukemia, sister melanoma | No pertinent past medical hx. | Invasive Adenocarcinoma of lung, solid Acinar pattern | pT2a pN0pMX |
| 6 | 55 | Male | Caucasian | 27.48 | No Family hx noted | No pertinent past medical hx. | Adenocarcinoma, poorly differentiated | pT1b N0 |
| 7 | 61 | Female | Caucasian | 35.15 | Mother cancer (type not noted), Sister breast and lung cancer | No pertinent past medical hx. | moderately differentiated invasive keratinizing squamous cell carcinoma | pT2a N0 |
| 8 | 79 | Female | Caucasian | 26.17 | Grandmother Cancer (type not noted) | No pertinent past medical hx. | Non-necrotizing granulomatous | NA |
| 9 | 68 | Female | Caucasian | 28.71 | No Family hx noted | No pertinent past medical hx. | Invasive adenocarcinoma moderately differentiated | pT2a N0 |
| 10 | 81 | Male | Caucasian | 25.44 | No Family hx noted | No pertinent past medical hx. | Poorly differentiated squamous cell carcinoma, nonkeratinizing | IIIa, pT3 N1 |
