# Supplementary material for: Precision Oncology: Artificial Intelligence and DNA Methylation Analysis of Circulating Cell-Free DNA for Lung Cancer Detection
Source: Front Oncol. 2022 May 4;12:790645. doi: 10.3389/fonc.2022.790645 (PMC9114890; doi:10.3389/fonc.2022.790645)
Supplement: Supplementary file 6 [file Table_4.docx]

Supplemental Table S4: Lung Cancer Prediction after controlling for age and gender using a 50-marker algorithm.

Results of cf-DNA Lung Cancer with demographics (CV 50 Variables)

|  | SVM | GLM | PAM | RF | LDA | DL |
| --- | --- | --- | --- | --- | --- | --- |
| AUC  95% CI | 1.0000  (0.9000-1) | 1.0000  (0.9000-1) | 0.9855  (0.8900-1) | 1.0000  (0.9000-1) | 1.0000  (0.9000-1) | 1.0000  (0.9500-1) |
| Sensitivity | 0.9500 | 0.9700 | 0.9600 | 0.9800 | 0.9300 | 1.0000 |
| Specificity | 1.0000 | 1.0000 | 1.0000 | 1.0000 | 1.0000 | 1.0000 |

Results of cf-DNA Lung Cancer with demographics (Bootstrapping 50 Variables)

|  | SVM | GLM | PAM | RF | LDA | DL |
| --- | --- | --- | --- | --- | --- | --- |
| AUC  95% CI | 1.0000  (0.9000-1) | 1.0000  (0.9000-1) | 0.9877  (0.9000-1) | 1.0000  (0.9000-1) | 1.0000  (0.9000-1) | 1.0000  (0.9500-1) |
| Sensitivity | 0.9600 | 0.9800 | 0.9700 | 0.9800 | 0.9400 | 1.0000 |
| Specificity | 1.0000 | 1.0000 | 1.0000 | 1.0000 | 1.0000 | 1.0000 |

Important predictors for DL in decreasing order:

DL: cg21466229, cg00071702, cg01174674, cg07618979, cg15684274, cg11107657, cg16550438, cg21656251, cg06216400, cg19343034, cg25167447, cg07240877, cg19316489, cg17266153, cg26982323, cg00066184, cg17382950, cg22681114, cg18486815, cg09232520, cg17647091, cg13151361, cg08855953, cg03387050, cg24156949, cg11934849, cg15955410, cg24043861, cg20727403, cg27199414, cg11270834, cg02401627, cg19461907, cg26372202, cg24072640, cg08259526, cg26832183, cg26420606, cg06831761, cg21757872, cg15119683, cg02484127, cg09607178, cg23957525, cg15626105, cg17412632, cg14371649, cg16993797, cg02080857, cg00823789
