# Supplementary material for: Precision Oncology: Artificial Intelligence and DNA Methylation Analysis of Circulating Cell-Free DNA for Lung Cancer Detection
Source: Front Oncol. 2022 May 4;12:790645. doi: 10.3389/fonc.2022.790645 (PMC9114890; doi:10.3389/fonc.2022.790645)
Supplement: Supplementary file 7 [file Table_5.docx]

Supplemental Table S5: Function of genes found to be epigenetically altered and significantly enriched in Gene Enrichment Analysis

| Gene | Gene Function | Reference |
| --- | --- | --- |
| *AKT2* and *AKT3* | belong to protein kinase family and  *- AKT2* involved in cell survival mechanism as seen in NSCLC cells - AKT3 controls the cell-cycle progression by phosphorylation activity in lung adenocarcinomas | (Lee et al., 2011)  (Laliotis et al., 2021) |
| *CDKN1C* and *CDKN2A* | cyclin dependent kinase inhibitors and associated with chemoresistance of SCLC and smoking related NSCLC | (Tam et al., 2013) (Qiu et al., 2019) |
| *BIRC3* | gene has antiapoptotic role in cancer cells | (Frazzi, 2021) |
| *CCND1* | was found to be overexpressed in tumor cells | (Gautschi et al., 2007) |
| CDK4 and CDK6 | increases signaling process in cancer cells by phosphorylating retinoblastoma protein that results in cell transition from G_1_ to S phase of cell cycle | (Pacheco and Schenk, 2019) |
| *FHIT, FN1, FOXO3* and *GRB2* | regulates epithelial-mesenchymal transition and/or metastasis and associated with LC | (Suh et al., 2014;Xu et al., 2016;Li et al., 2019;Jiramongkol and Lam, 2020) |
| *ITGA2, ITGA3* and *ITGA6* | integrin coding genes that participate in cell adhesion, proliferation, and differentiation  -have anti-cancer properties identified in LC | (AKSORN and CHANVORACHOTE, 2019) |
| *KRAS* | a RAS family proto-oncogene involved in complex mechanism to regulate multiple canonical signaling pathways that regulate cellular functions such as cell motility, proliferation*,* survival, and apoptosis, associated with LC | (Román et al., 2018) |
| RASSF5 | mediates Ras-dependent programmed cell death and regulates cell growth | (Zhou et al., 2014) |
| *NOS2* | enhances KRAS induced LC transformation | (Okayama et al., 2013) |
| *LAMA2, LAMA4, LAMB2* and *LAMC2* | laminins, they are involved in cell signaling and metastasis | (Maltseva and Rodin, 2018) |
| MYC | Promotes metastasis in NSCLC | (Rapp et al., 2009) |
| *MAPK1* | plays a significant role in inducing programmed cell death protein in lung adenocarcinoma cells | (Stutvoet et al., 2019) |
| *PIAS3*, *PIK3CA, PLCG2, PTK2* | mutations and differential expression of these genes commonly found in NSCLC | (Kluge et al., 2011)  (Scheffler et al., 2015)  (Tong et al., 2019;Qian et al., 2020) |
| *RARB* | Gene hypermethylation is commonly identified in NSCLC | (Li et al., 2017) |
| *RB1* | Regulation of cell cycle and response to chemotherapy, also mutations common in SCLC and seen in a minority of NSCLC | (Bhateja et al., 2019) |
| LC and *PTEN* | promotes G0/G1 arrest and cell apoptosis and inhibits NSCLC | (Liu et al., 2019) |

**References:**

Aksorn, N., and Chanvorachote, P. (2019). Integrin as a Molecular Target for Anti-cancer Approaches in Lung Cancer. *Anticancer Research* 39**,** 541-548.

Bhateja, P., Chiu, M., and Wildey, G. (2019). Retinoblastoma mutation predicts poor outcomes in advanced non small cell lung cancer. 8**,** 1459-1466.

Frazzi, R. (2021). BIRC3 and BIRC5: multi‐faceted inhibitors in cancer. *Cell & Bioscience* 11**,** 8.

Gautschi, O., Ratschiller, D., Gugger, M., Betticher, D.C., and Heighway, J. (2007). Cyclin D1 in non-small cell lung cancer: A key driver of malignant transformation. *Lung Cancer* 55**,** 1-14.

Jiramongkol, Y., and Lam, E.W.F. (2020). FOXO transcription factor family in cancer and metastasis. *Cancer metastasis reviews* 39**,** 681-709.

Kluge, A., Dabir, S., Vlassenbroeck, I., Eisenberg, R., and Dowlati, A. (2011). Protein inhibitor of activated STAT3 expression in lung cancer. *Mol Oncol* 5**,** 256-264.

Laliotis, G.I., Chavdoula, E., Paraskevopoulou, M.D., Kaba, A., La Ferlita, A., Singh, S., Anastas, V., Nair, K.A., 2nd, Orlacchio, A., Taraslia, V., Vlachos, I., Capece, M., Hatzigeorgiou, A., Palmieri, D., Tsatsanis, C., Alaimo, S., Sehgal, L., Carbone, D.P., Coppola, V., and Tsichlis, P.N. (2021). AKT3-mediated IWS1 phosphorylation promotes the proliferation of EGFR-mutant lung adenocarcinomas through cell cycle-regulated U2AF2 RNA splicing. *Nature communications* 12**,** 4624-4624.

Lee, M.W., Kim, D.S., Lee, J.H., Lee, B.S., Lee, S.H., Jung, H.L., Sung, K.W., Kim, H.T., Yoo, K.H., and Koo, H.H. (2011). Roles of AKT1 and AKT2 in non-small cell lung cancer cell survival, growth, and migration. *Cancer Sci* 102**,** 1822-1828.

Li, B., Shen, W., Peng, H., Li, Y., Chen, F., Zheng, L., Xu, J., and Jia, L. (2019). Fibronectin 1 promotes melanoma proliferation and metastasis by inhibiting apoptosis and regulating EMT. *Onco Targets Ther* 12**,** 3207-3221.

Li, Y., Lu, D.-G., Ma, Y.-M., and Liu, H. (2017). Association between Retinoic acid receptor-β hypermethylation and NSCLC risk: a meta-analysis and literature review. *Oncotarget* 8**,** 5814-5822.

Liu, L., Huang, L., He, J., Cai, S., Weng, Y., Huang, S., and Ma, S. (2019). PTEN inhibits non‑small cell lung cancer cell growth by promoting G0/G1 arrest and cell apoptosis. *Oncol Lett* 17**,** 1333-1340.

Maltseva, D.V., and Rodin, S.A. (2018). [Laminins in Metastatic Cancer]. *Mol Biol (Mosk)* 52**,** 411-434.

Okayama, H., Saito, M., Oue, N., Weiss, J.M., Stauffer, J., Takenoshita, S., Wiltrout, R.H., Hussain, S.P., and Harris, C.C. (2013). NOS2 enhances KRAS-induced lung carcinogenesis, inflammation and microRNA-21 expression. *International journal of cancer* 132**,** 9-18.

Pacheco, J., and Schenk, E. (2019). CDK4/6 inhibition alone and in combination for non-small cell lung cancer. *Oncotarget* 10**,** 618-619.

Qian, D., Liu, H., Zhao, L., Wang, X., Luo, S., Moorman, P.G., Patz, E.F., Jr., Su, L., Shen, S., Christiani, D.C., and Wei, Q. (2020). Novel genetic variants in genes of the Fc gamma receptor-mediated phagocytosis pathway predict non-small cell lung cancer survival. *Translational lung cancer research* 9**,** 575-586.

Qiu, Z., Zhu, W., Meng, H., Tong, L., Li, X., Luo, P., Yi, L., Zhang, X., Guo, L., Wei, T., and Zhang, J. (2019). CDYL promotes the chemoresistance of small cell lung cancer by regulating H3K27 trimethylation at the CDKN1C promoter. *Theranostics* 9**,** 4717-4729.

Rapp, U.R., Korn, C., Ceteci, F., Karreman, C., Luetkenhaus, K., Serafin, V., Zanucco, E., Castro, I., and Potapenko, T. (2009). MYC is a metastasis gene for non-small-cell lung cancer. *PLoS One* 4**,** e6029.

Román, M., Baraibar, I., López, I., Nadal, E., Rolfo, C., Vicent, S., and Gil-Bazo, I. (2018). KRAS oncogene in non-small cell lung cancer: clinical perspectives on the treatment of an old target. *Molecular cancer* 17**,** 33-33.

Scheffler, M., Bos, M., Gardizi, M., König, K., Michels, S., Fassunke, J., Heydt, C., Künstlinger, H., Ihle, M., Ueckeroth, F., Albus, K., Serke, M., Gerigk, U., Schulte, W., Töpelt, K., Nogova, L., Zander, T., Engel-Riedel, W., Stoelben, E., Ko, Y.-D., Randerath, W., Kaminsky, B., Panse, J., Becker, C., Hellmich, M., Merkelbach-Bruse, S., Heukamp, L.C., Büttner, R., and Wolf, J. (2015). PIK3CA mutations in non-small cell lung cancer (NSCLC): genetic heterogeneity, prognostic impact and incidence of prior malignancies. *Oncotarget* 6**,** 1315-1326.

Stutvoet, T.S., Kol, A., De Vries, E.G., De Bruyn, M., Fehrmann, R.S., Terwisscha Van Scheltinga, A.G., and De Jong, S. (2019). MAPK pathway activity plays a key role in PD-L1 expression of lung adenocarcinoma cells. *The Journal of pathology* 249**,** 52-64.

Suh, S.S., Yoo, J.Y., Cui, R., Kaur, B., Huebner, K., Lee, T.K., Aqeilan, R.I., and Croce, C.M. (2014). FHIT suppresses epithelial-mesenchymal transition (EMT) and metastasis in lung cancer through modulation of microRNAs. *PLoS Genet* 10**,** e1004652.

Tam, K.W., Zhang, W., Soh, J., Stastny, V., Chen, M., Sun, H., Thu, K., Rios, J.J., Yang, C., Marconett, C.N., Selamat, S.A., Laird-Offringa, I.A., Taguchi, A., Hanash, S., Shames, D., Ma, X., Zhang, M.Q., Lam, W.L., and Gazdar, A. (2013). CDKN2A/p16 inactivation mechanisms and their relationship to smoke exposure and molecular features in non-small-cell lung cancer. *J Thorac Oncol* 8**,** 1378-1388.

Tong, X., Tanino, R., Sun, R., Tsubata, Y., Okimoto, T., Takechi, M., and Isobe, T. (2019). Protein tyrosine kinase 2: a novel therapeutic target to overcome acquired EGFR-TKI resistance in non-small cell lung cancer. *Respiratory Research* 20**,** 270.

Xu, L.J., Wang, Y.C., Lan, H.W., Li, J., and Xia, T. (2016). Grb2-associated binder-2 gene promotes migration of non-small cell lung cancer cells via Akt signaling pathway. *American journal of translational research* 8**,** 1208-1217.

Zhou, X.H., Yang, C.Q., Zhang, C.L., Gao, Y., Yuan, H.B., and Wang, C. (2014). RASSF5 inhibits growth and invasion and induces apoptosis in osteosarcoma cells through activation of MST1/LATS1 signaling. *Oncol Rep* 32**,** 1505-1512.
