# Supplementary material for: Precision Oncology: Artificial Intelligence and DNA Methylation Analysis of Circulating Cell-Free DNA for Lung Cancer Detection
Source: Front Oncol. 2022 May 4;12:790645. doi: 10.3389/fonc.2022.790645 (PMC9114890; doi:10.3389/fonc.2022.790645)
Supplement: Supplementary file 11 [file Table_9.docx]

Supplemental Table S9: Function of long non-coding RNA genes found to be epigenetically altered in lung cancer

| **lncRNA name** | **Function relevant to lung cancer** | **References** |
| --- | --- | --- |
| ADD3-AS1 | ADD3-AS1 is a RET fusion partner identified in lung adenocarcinoma | (Zhang et al., 2019) |
| ARHGEF26-AS1 | ARHGEF26-AS1 upregulation is predicted to be associated with overall survival rate in lung cancer cases | (Huang and Huang, 2021) |
| B4GALT4-AS1 | B4GALT1-AS1 acted as a lncRNA that drives tumor progression in NSCLC via the regulation of the miR-30e/SOX9 axis | (Lin et al., 2020) |
| CASC15 | CASC15 promotes lung cancer through the miR-766-5p/KLK12 axis | (Bai et al., 2019) |
| CYP1B1-AS1 | CYP1B1-AS1 is predicted to show redox-related prognostic signature | (Ren et al., 2021) |
| DARS-AS1 | DARS-AS1 targets miR-532-3p and promotes tumorigenesis of non-small cell lung cancer | (Liu et al., 2021) |
| DGCR5 | DGCR5 was downregulated in the serum and tissues of lung cancer patients and was associated with poor prognosis. | (Chen et al., 2017) |
| DISC1-IT1 | DISC1-IT1 is correlated with overall survival rate in lung adenocarcinoma | (Wu et al., 2020) |
| HAS2-AS1 | HAS2-AS1 was found to be up-regulated, which, in turn, indicated the poor prognosis of NSCLC patients | (Sun et al., 2020) |
| LEF1-AS1 | LEF1-AS1 was found to be upregulated in lung cancer tissues, LEF1-AS1 promotes malignancy by targeting miR489/SOX4 axis in NSCLC and also regulates miR-544a/FOXP1 axis in lung cancer | (Yang et al., 2019) (Wang et al., 2019) |
| LIFR-AS1 | LIFR-AS1 is involved in invasion and metastasis by targeting miR-942-5p/ZNF471 axis in NSCLC | (Wang et al., 2020) |
| LINC00665 | LINC00665 functions as a competing endogenous RNA for miR-98 and thus activates downstream AKR1B10-ERK signaling and promotes lung adenocarcinoma progression | (Cong et al., 2019) |
| LINC00858 | LINC00858 functions as a competitive endogenous RNA for miR-422a and modulates KLK4 and leads to tumor progression in NSCLC | (Zhu et al., 2017) |
| LINC00887 | LINC00887 in upregulated state can accelerate the malignant transformation ability of NSCLC cells by degrading miRNAs, possibly miR-613, miR-206 and miR-1-2 | (Tian et al., 2019) |
| LINC01192 | LINC01192 showed higher expression in lung squamous cell carcinoma, further functional role is yet to be understood | (Liu et al., 2017) |

References:

Bai, Y., Zhang, G., Cheng, R., Yang, R., and Chu, H. (2019). CASC15 contributes to proliferation and invasion through regulating miR-766-5p/ KLK12 axis in lung cancer. *Cell Cycle* 18**,** 2323-2331.

Chen, E.G., Zhang, J.S., Xu, S., Zhu, X.J., and Hu, H.H. (2017). Long non-coding RNA DGCR5 is involved in the regulation of proliferation, migration and invasion of lung cancer by targeting miR-1180. *Am J Cancer Res* 7**,** 1463-1475.

Cong, Z., Diao, Y., Xu, Y., Li, X., Jiang, Z., Shao, C., Ji, S., Shen, Y., De, W., and Qiang, Y. (2019). Long non-coding RNA linc00665 promotes lung adenocarcinoma progression and functions as ceRNA to regulate AKR1B10-ERK signaling by sponging miR-98. *Cell Death Dis* 10**,** 84.

Huang, Y.J., and Huang, C.J. (2021). Construction of a 5 immune-related lncRNA-based prognostic model of NSCLC via bioinformatics. 100**,** e27222.

Lin, J.H., Chen, F.N., Wu, C.X., Hu, S.Q., and Ma, J. (2020). Long non-coding RNA B4GALT1-Antisense RNA 1/microRNA-30e/SRY-box transcription factor 9 signaling axis contributes to non-small cell lung cancer cell growth. *Oncol Lett* 20**,** 284.

Liu, B., Chen, Y., and Yang, J. (2017). LncRNAs are altered in lung squamous cell carcinoma and lung adenocarcinoma. *Oncotarget* 8**,** 24275-24291.

Liu, D., Liu, H., Jiang, Z., Chen, M., and Gao, S. (2021). Long non-coding RNA DARS-AS1 promotes tumorigenesis of non-small cell lung cancer via targeting miR-532-3p. *Minerva Med* 112**,** 408-409.

Ren, J., Wang, A., Liu, J., and Yuan, Q. (2021). Identification and validation of a novel redox-related lncRNA prognostic signature in lung adenocarcinoma. *Bioengineered* 12**,** 4331-4348.

Sun, P., Sun, L., Cui, J., Liu, L., and He, Q. (2020). Long noncoding RNA HAS2-AS1 accelerates non-small cell lung cancer chemotherapy resistance by targeting LSD1/EphB3 pathway. *Am J Transl Res* 12**,** 950-958.

Tian, Y., Yu, M., Sun, L., Liu, L., Huo, S., Shang, W., Sheng, S., Wang, J., Sun, J., Hu, Q., Dou, Y., Zhu, J., Ren, X., and Yang, S. (2019). Long non‑coding RNA00887 reduces the invasion and metastasis of non‑small cell lung cancer by causing the degradation of miRNAs. *Oncol Rep* 42**,** 1173-1182.

Wang, A., Zhao, C., and Gao, Y. (2019). LEF1-AS1 contributes to proliferation and invasion through regulating miR-544a/ FOXP1 axis in lung cancer. 37**,** 1127-1134.

Wang, Q., Wu, J., Huang, H., Jiang, Y., Huang, Y., Fang, H., Zheng, G., Zhou, X., Wu, Y., Lei, C., and Hu, D. (2020). lncRNA LIFR-AS1 suppresses invasion and metastasis of non-small cell lung cancer via the miR-942-5p/ZNF471 axis. 20**,** 180.

Wu, X., Sui, Z., Zhang, H., Wang, Y., and Yu, Z. (2020). Integrated Analysis of lncRNA-Mediated ceRNA Network in Lung Adenocarcinoma. *Front Oncol* 10**,** 554759.

Yang, J., Lin, X., Jiang, W., Wu, J., and Lin, L. (2019). lncRNA LEF1-AS1 Promotes Malignancy in Non-Small-Cell Lung Cancer by Modulating the miR-489/SOX4 Axis. *DNA Cell Biol* 38**,** 1013-1021.

Zhang, K., Chen, H., Wang, Y., Yang, L., Zhou, C., Yin, W., Wang, G., Mao, X., Xiang, J., Li, B., Zhang, T., and Fei, S. (2019). Clinical Characteristics and Molecular Patterns of RET-Rearranged Lung Cancer in Chinese Patients. *Oncol Res* 27**,** 575-582.

Zhu, S.P., Wang, J.Y., Wang, X.G., and Zhao, J.P. (2017). Long intergenic non-protein coding RNA 00858 functions as a competing endogenous RNA for miR-422a to facilitate the cell growth in non-small cell lung cancer. *Aging (Albany NY)* 9**,** 475-486.
