# Supplementary material for: Precision Oncology: Artificial Intelligence and DNA Methylation Analysis of Circulating Cell-Free DNA for Lung Cancer Detection
Source: Front Oncol. 2022 May 4;12:790645. doi: 10.3389/fonc.2022.790645 (PMC9114890; doi:10.3389/fonc.2022.790645)
Supplement: Supplementary file 12 [file Table_10.docx]

Supplemental Table S10: Known or Putative Roles of Biomarker (CpG) Genes in Cancer and assessed by AI to be top lung cancer markers

| Gene | Roles | Reference |
| --- | --- | --- |
| *TEAD1* | -codes for a transcription factor that plays a key role in organ size control and tumor suppression - associated with lung cancer | (Yu et al., 2018;Huh et al., 2019) |
| *ERLIN2* | regulates cell cycle progression | (Wang et al., 2012) |
| *DNAJC10* | antioncogenic properties and downregulation of gene has been identified in various cancer types | (Acun and Senses, 2020) |
| *MCAM* | involved in cell adhesion, cell signaling, cell migration and proliferation in association with NSCLC | (Kristiansen et al., 2003) |
| MEIS3 | regulates cell survival by targeting 3-phosphoinositide-dependent protein kinase 1 which is involved in PI3K-Akt signaling pathway | (Liu et al., 2010) |
| *BTRC* | interacts with *TSPAN15* and triggers the transcription of many metastasis associated genes | (Zhang et al., 2018) |
| *DUXAP10* | a pseudogene (long noncoding RNA), functions are unclear. Probably epigenetically represses LATS2 and RRAD expression and may exert oncogenic role in NSCLC | (Wei et al., 2017) |
| *CDH4* | decreases cell migration and invasion | (Gao et al., 2016) |
| *MCPH1* | elevated expression represses uncontrolled cell growth via apoptosis in LC | (Zhou et al., 2016) |
| *NCOA2* | suppress cell proliferation by inducing cell cycle arrest and apoptosis probably by downregulating MAPK/ERK signaling | (Cai et al., 2019) |
| *SAFB2* | an estrogen receptor corepressor, elevated expression inhibits cell proliferation | (Townson et al., 2003) |
| *PTPN11* | involved in signal transduction of growth factors, integrin receptors and cytokines and plays a vital role in oncogenic signaling pathways | (Ruess et al., 2018) |
| *DLK2* | gene interacts with *NOTCH1* and modulates NOTCH signaling mediated cell proliferation | (Sánchez-Solana et al., 2011) |
| ULK1 | represses autophagy induction based on response to various environmental influence such as inhibitors | (Martin et al., 2018) |
| *SRPK2* | regulates cell cycle process and found to be upregulated in NSCLC tissue | (Li et al., 2019) |
| *KCNQ1DN* | higher expression suppressed xenograft tumors and corresponding c-Myc in renal cell cancer. Has not been studied in association with LC | (Yang et al., 2019) |
| *AK7* | differential expression of AK7 previously observed in ovarian cancer | (Zhang et al., 2021) |
| *ODZ2 / TENM2* | correlated with survival in ovarian cancer | (Graumann et al., 2017) |
| *DCDC2* | promotes cell migration and induces resistance to microtubule targeting medicines | (Longoni et al., 2013) |
| *USP36* | plays a role in promoting cell proliferation and survival by regulating c-Myc levels | (Sun et al., 2015) |
| *NFATC2* | Associated with (poor) tumor differentiation in LC and correlates with overall survival in LC patients | (Xiao et al., 2017) |
| *NOC2L* | expression inhibits tumor growth | (Wang et al., 2019) |
| *PARK2 and* WNK2 | gene mutations identified in lung tumors and both genes have tumor and/or cell growth suppressing activity | (Jun et al., 2009;Xiong et al., 2015) |
| *GNAL* | has tumorigenesis properties | (Yi et al., 2009) |

**References:**

Acun, T., and Senses, K.M. (2020). Downregulation of DNAJC10 (ERDJ5) is associated with poor survival in breast cancer. *Breast Cancer* 27**,** 483-489.

Cai, M., Liang, X., Sun, X., Chen, H., Dong, Y., Wu, L., Gu, S., and Han, S. (2019). Nuclear Receptor Coactivator 2 Promotes Human Breast Cancer Cell Growth by Positively Regulating the MAPK/ERK Pathway. *Front Oncol* 9**,** 164.

Gao, Y., Wang, G., Zhang, C., Lin, M., Liu, X., Zeng, Y., and Liu, J. (2016). Long non-coding RNA linc-cdh4-2 inhibits the migration and invasion of HCC cells by targeting R-cadherin pathway. *Biochem Biophys Res Commun* 480**,** 348-354.

Graumann, R., Di Capua, G.A., Oyarzun, J.E., Vasquez, M.A., Liao, C., Branes, J.A., Roa, I., Casanello, P., Corvalan, A.H., Owen, G.I., Delgado, I., Zangemeister-Wittke, U., and Ziegler, A. (2017). Expression of teneurins is associated with tumor differentiation and patient survival in ovarian cancer. *PLoS One* 12**,** e0177244.

Huh, H.D., Kim, D.H., Jeong, H.-S., and Park, H.W. (2019). Regulation of TEAD Transcription Factors in Cancer Biology. *Cells* 8**,** 600.

Jun, P., Hong, C., Lal, A., Wong, J.M., Mcdermott, M.W., Bollen, A.W., Plass, C., Held, W.A., Smiraglia, D.J., and Costello, J.F. (2009). Epigenetic silencing of the kinase tumor suppressor WNK2 is tumor-type and tumor-grade specific. *Neuro Oncol* 11**,** 414-422.

Kristiansen, G., Yu, Y., Schlüns, K., Sers, C., Dietel, M., and Petersen, I. (2003). Expression of the cell adhesion molecule CD146/MCAM in non-small cell lung cancer. *Anal Cell Pathol* 25**,** 77-81.

Li, X., Yang, S., Zhang, M., Xie, S., and Xie, Z. (2019). Downregulation of SRPK2 promotes cell cycle arrest though E2F1 in non-small cell lung cancer. *Eur J Histochem* 63.

Liu, J., Wang, Y., Birnbaum, M.J., and Stoffers, D.A. (2010). Three-amino-acid-loop-extension homeodomain factor Meis3 regulates cell survival via PDK1. *Proc Natl Acad Sci U S A* 107**,** 20494-20499.

Longoni, N., Kunderfranco, P., Pellini, S., Albino, D., Mello-Grand, M., Pinton, S., D'ambrosio, G., Sarti, M., Sessa, F., Chiorino, G., Catapano, C.V., and Carbone, G.M. (2013). Aberrant expression of the neuronal-specific protein DCDC2 promotes malignant phenotypes and is associated with prostate cancer progression. *Oncogene* 32**,** 2315-2324, 2324 e2311-2314.

Martin, K.R., Celano, S.L., Solitro, A.R., Gunaydin, H., Scott, M., O'hagan, R.C., Shumway, S.D., Fuller, P., and Mackeigan, J.P. (2018). A Potent and Selective ULK1 Inhibitor Suppresses Autophagy and Sensitizes Cancer Cells to Nutrient Stress. *iScience* 8**,** 74-84.

Ruess, D.A., Heynen, G.J., Ciecielski, K.J., Ai, J., Berninger, A., Kabacaoglu, D., Gorgulu, K., Dantes, Z., Wormann, S.M., Diakopoulos, K.N., Karpathaki, A.F., Kowalska, M., Kaya-Aksoy, E., Song, L., Van Der Laan, E.a.Z., Lopez-Alberca, M.P., Nazare, M., Reichert, M., Saur, D., Erkan, M.M., Hopt, U.T., Sainz, B., Jr., Birchmeier, W., Schmid, R.M., Lesina, M., and Algul, H. (2018). Mutant KRAS-driven cancers depend on PTPN11/SHP2 phosphatase. *Nat Med* 24**,** 954-960.

Sánchez-Solana, B., Nueda, M.L., Ruvira, M.D., Ruiz-Hidalgo, M.J., Monsalve, E.M., Rivero, S., García-Ramírez, J.J., Díaz-Guerra, M.J.M., Baladrón, V., and Laborda, J. (2011). The EGF-like proteins DLK1 and DLK2 function as inhibitory non-canonical ligands of NOTCH1 receptor that modulate each other's activities. *Biochimica et Biophysica Acta (BBA) - Molecular Cell Research* 1813**,** 1153-1164.

Sun, X.-X., He, X., Yin, L., Komada, M., Sears, R.C., and Dai, M.-S. (2015). The nucleolar ubiquitin-specific protease USP36 deubiquitinates and stabilizes c-Myc. *Proceedings of the National Academy of Sciences* 112**,** 3734-3739.

Townson, S.M., Dobrzycka, K.M., Lee, A.V., Air, M., Deng, W., Kang, K., Jiang, S., Kioka, N., Michaelis, K., and Oesterreich, S. (2003). SAFB2, a new scaffold attachment factor homolog and estrogen receptor corepressor. *J Biol Chem* 278**,** 20059-20068.

Wang, G., Liu, G., Wang, X., Sethi, S., Ali-Fehmi, R., Abrams, J., Zheng, Z., Zhang, K., Ethier, S., and Yang, Z.Q. (2012). ERLIN2 promotes breast cancer cell survival by modulating endoplasmic reticulum stress pathways. *BMC Cancer* 12**,** 225.

Wang, Q., Ding, J., Nan, G., Lyu, Y., and Ni, G. (2019). LncRNA NOC2L-4.1 functions as a tumor oncogene in cervical cancer progression by regulating the miR-630/YAP1 pathway. *J Cell Biochem* 120**,** 16913-16920.

Wei, C.C., Nie, F.Q., Jiang, L.L., Chen, Q.N., Chen, Z.Y., Chen, X., Pan, X., Liu, Z.L., Lu, B.B., and Wang, Z.X. (2017). The pseudogene DUXAP10 promotes an aggressive phenotype through binding with LSD1 and repressing LATS2 and RRAD in non small cell lung cancer. *Oncotarget* 8**,** 5233-5246.

Xiao, Z.J., Liu, J., Wang, S.Q., Zhu, Y., Gao, X.Y., Tin, V.P., Qin, J., Wang, J.W., and Wong, M.P. (2017). NFATc2 enhances tumor-initiating phenotypes through the NFATc2/SOX2/ALDH axis in lung adenocarcinoma. *Elife* 6.

Xiong, D., Wang, Y., Kupert, E., Simpson, C., Pinney, S.M., Gaba, C.R., Mandal, D., Schwartz, A.G., Yang, P., De Andrade, M., Pikielny, C., Byun, J., Li, Y., Stambolian, D., Spitz, M.R., Liu, Y., Amos, C.I., Bailey-Wilson, J.E., Anderson, M., and You, M. (2015). A recurrent mutation in PARK2 is associated with familial lung cancer. *Am J Hum Genet* 96**,** 301-308.

Yang, F., Wu, Q., Zhang, L., Xie, W., Sun, X., Zhang, Y., Wang, L., Dai, Q., Yu, H., Chen, Q., Sheng, H., Qiu, J., He, X., Miao, H., He, F., and Zhang, K. (2019). The long noncoding RNA KCNQ1DN suppresses the survival of renal cell carcinoma cells through downregulating c-Myc. *J Cancer* 10**,** 4662-4670.

Yi, C.H., Zheng, T., Leaderer, D., Hoffman, A., and Zhu, Y. (2009). Cancer-related transcriptional targets of the circadian gene NPAS2 identified by genome-wide ChIP-on-chip analysis. *Cancer Lett* 284**,** 149-156.

Yu, M., Chen, Y., Li, X., Yang, R., Zhang, L., Huangfu, L., Zheng, N., Zhao, X., Lv, L., Hong, Y., Liang, H., and Shan, H. (2018). YAP1 contributes to NSCLC invasion and migration by promoting Slug transcription via the transcription co-factor TEAD. *Cell Death & Disease* 9**,** 464.

Zhang, B., Zhang, Z., Li, L., Qin, Y.R., Liu, H., Jiang, C., Zeng, T.T., Li, M.Q., Xie, D., Li, Y., Guan, X.Y., and Zhu, Y.H. (2018). TSPAN15 interacts with BTRC to promote oesophageal squamous cell carcinoma metastasis via activating NF-kappaB signaling. *Nat Commun* 9**,** 1423.

Zhang, X.Y., Zhou, L.L., Jiao, Y., Li, Y.Q., Guan, Y.N., Zhao, Y.C., and Zheng, L.W. (2021). Adenylate kinase 7 is a prognostic indicator of overall survival in ovarian cancer. *Medicine (Baltimore)* 100**,** e24134.

Zhou, L., Bai, Y., Li, Y., Liu, X., Tan, T., Meng, S., He, W., Wu, X., and Dong, Z. (2016). Overexpression of MCPH1 inhibits uncontrolled cell growth by promoting cell apoptosis and arresting the cell cycle in S and G2/M phase in lung cancer cells. *Oncol Lett* 11**,** 365-372.
